# Safety considerations and clinical monitoring during pulsed field ablation

**DOI:** 10.1093/europace/euag248

**Published:** 2026-10-21

**Authors:** Pascal Defaye, Edward P Gerstenfeld, Melanie A Gunawardene, Jose L Merino, Jose M Guerra, Sandrine Venier, Savannah Bifulco, Andrea Sarkozy

**Affiliations:** Arrhythmia Department/Cardiology Department, University Hospital of Grenoble Alpes, Avenue des Maquis du Grésivaudan, CS 10217, Grenoble 38043, France; Electrophysiology Section, Division of Cardiology, Department of Medicine, University of California San Francisco, 500 Parnassus Ave, San Francisco, CA 94143, USA; CCB, MVZ CCB Frankfurt und Main Taunus GbR, Markus Hospital, Wilhelm Epstein Strasse 4, Frankfurt am Main 60431, Germany; Centre for Heart Rhythm Disorders, Adelaide University and Royal Adelaide Hospital, Adelaide, SA, Australia; University Hospital Giessen, Department of Cardiology, Giessen, Germany; Arrhythmia and Robotic Electrophysiology Unit, La Paz University Hospital, IdiPaz, Madrid, Spain; Hospital Memorial Publio Cordón, Department of Cardiology, Pozuelo Alarcón, Madrid, Spain; Hospital de la Santa Creu i Sant Pau, Institut de Recerca Sant Pau (IR SANT PAU), CIBERCV, Universitat Autònoma de Barcelona, Barcelona, Spain; Arrhythmia Department/Cardiology Department, University Hospital of Grenoble Alpes, Avenue des Maquis du Grésivaudan, CS 10217, Grenoble 38043, France; Department of Electrophysiology, Boston Scientific, 4100 Hamline Ave N, St Paul, MN 55112, USA; Heart Rhythm Management Centre, Postgraduate Program in Cardiac Electrophysiology and Pacing, Universitair Ziekenhuis Brussel, Vrije Universiteit Brussel, European Reference Networks Guard-Heart, Brussels, Belgium

**Keywords:** Coronary spasm, Embolic effects, Microbubbles, Haemolysis, CIED interactions, Pulsed field ablation, Coronary injury, Phrenic nerve stunning, Irreversible Electroporation, Atrial fibrillation ablation, Safety profile, Coronary vasospasm, Coronary artery spasm, Acute kidney injury, Silent cerebral lesions, Microembolism, Thromboembolic events, Electromagnetic interference, Catheter design, Procedural complications, Risk mitigation, Electrophysiology

## Abstract

Pulsed field ablation (PFA) has emerged as a transformative technology in cardiac electrophysiology, offering a potentially superior safety profile compared with thermal ablation due to its non-thermal mechanism of irreversible electroporation. Clinical experience confirms a substantial reduction in traditional complications such as pulmonary vein stenosis, atrio-oesophageal fistula, and permanent phrenic nerve injury, though PFA introduces a distinct spectrum of physiologic effects requiring consideration. Coronary vasospasm is the most characteristic phenomenon, ranging from transient, subclinical constriction to rare ischaemic or arrhythmic events; mitigation relies on coronary anatomical awareness, optimized lesion placement, and prophylactic vasodilators. Haemolysis, caused by red blood cell electroporation, varies with catheter design and pulse burden and is typically mild, though high exposure and inadequate hydration may rarely lead to acute kidney injury. Silent cerebral lesions could result from procedural and/or energy-related factors, including gas formation, air ingress, catheter manipulation, and anticoagulation management, with uncertain long-term clinical impact but opportunities for technique-driven reduction. Interactions between PFA and cardiac implantable electronic devices are system- and context-dependent, with monopolar configurations and catheter-lead proximity influencing the risk of transient interference and rare permanent damage.

## Graphical Abstract

**Graphical Abstract:**
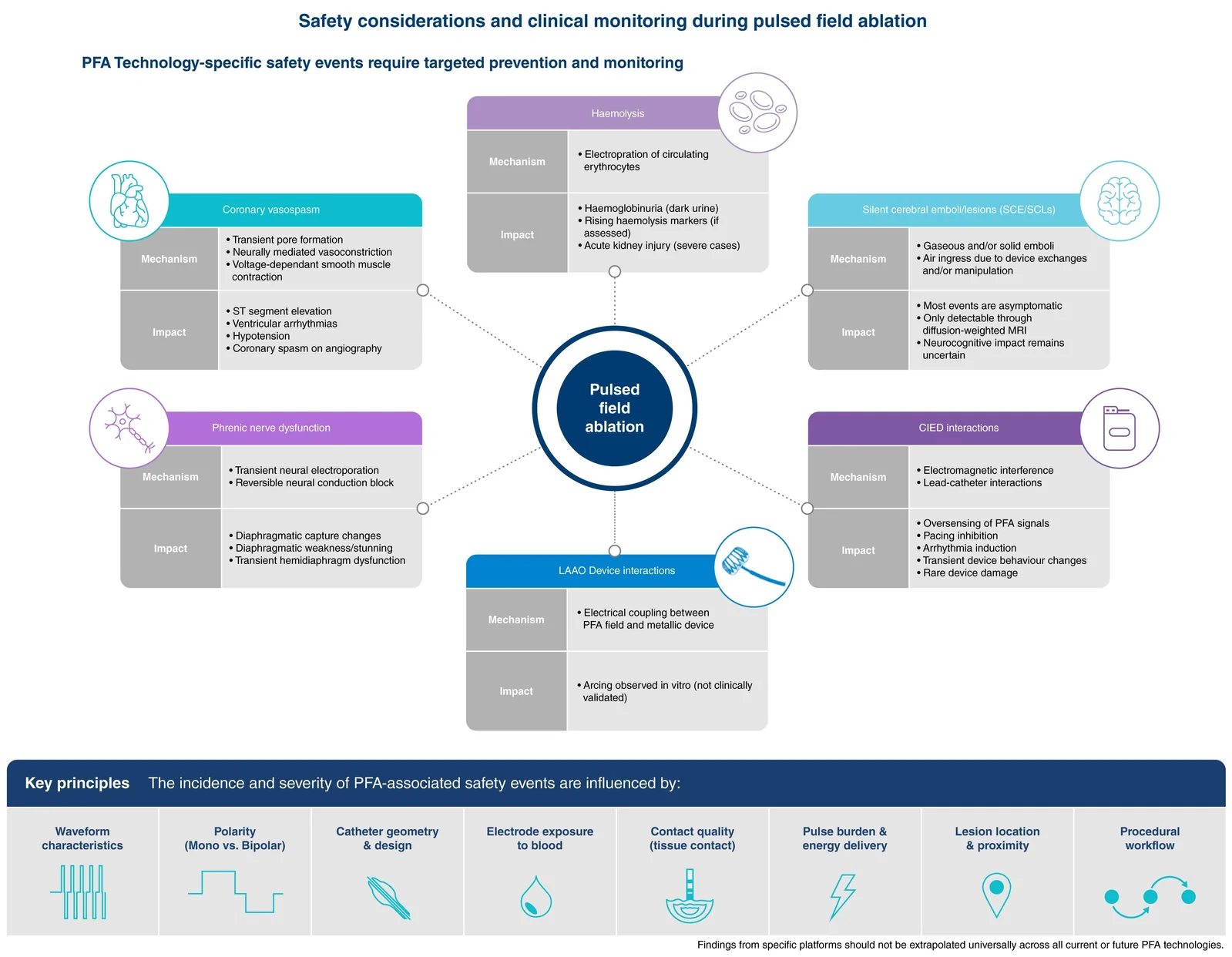
Overview of safety considerations during pulsed field ablation. Diagram illustrating major PFA-specific safety events, including coronary vasospasm, haemolysis, silent cerebral emboli/lesions, CIED interactions, LAAO device interactions, and phrenic nerve dysfunction, together with their mechanisms, clinical consequences, and key factors influencing event risk and severity.

## Introduction

One of the main advantages of pulsed field ablation is its potentially superior safety profile compared to thermal technologies.^1^ The fundamentally different mechanisms of action of PFA, based on the non-thermal phenomenon of electroporation (IRE), have substantially reduced the risk of serious adverse events typically associated with thermal ablation technologies, such as pulmonary vein (PV) stenosis, atrio-oesophageal fistula (AEF), and permanent phrenic nerve injury (PNI).^2,3^ Accumulated clinical evidence from large real-world patient populations has virtually confirmed the near complete elimination of some of these adverse events with occurrence rates approaching zero.^4,5^ However, PFA has also revealed several relevant novel adverse effects that deserve special attention.

The main distinctive characteristic of PFA is the use of high-intensity electric fields, several orders of magnitude higher in intensity and shorter in duration than the field applied in radiofrequency (RF) ablation, to induce IRE in the target cardiac tissue. These high-intensity electric fields may give rise to a specific set of adverse effects.

Perhaps the most prominent technology-specific effect associated with PFA relates to its effects on coronary arteries, particularly acute effects leading to coronary artery spasm. Other observed coronary events, such as delayed spasm, remain incompletely understood, and their link to PFA energy has yet to be clearly established. The second safety event warranting attention is haemolysis associated with the application of high-intensity electric fields within the blood pool and the rarely observed ensuing acute kidney injury (AKI). Lastly, thromboembolic events, although not unique to PFA procedures, deserve special consideration, as the underlying mechanisms in PFA may differ from those observed with RF ablation.

Although the incidence rates of some of these events are very low and the clinical relevance of others remains apparently limited, it is important to stay vigilant and understand the mechanism underlying their occurrence. This article summarizes the current scientific evidence regarding the principal mechanisms underlying the main PFA technology-specific effects, as well as potential mitigation and patient management strategies. Additionally, a direct comparison with thermal technologies is discussed to provide a clearer understanding of the main differences between technologies.

## Coronary effects

### Pathophysiology of acute coronary spasm

Pulsed field ablation–induced vasospasm is a long-recognized vascular phenomenon extensively characterized in multiple non-coronary tissues.^6^ Foundational work in skeletal muscle, the microvasculature, neural pathways, and epithelial barriers consistently describes a reproducible electroporation-related vasomotor response involving immediate neurally mediated smooth-muscle contraction, transient endothelial pore formation, delayed microvascular constriction, vascular lock, increased vessel permeability, and red blood cell (RBC) stacking.^7–9^ This experimental framework constitutes the primary foundation for understanding vascular responses to PFA.

By contrast, our coronary-specific understanding remains considerably more limited. Early descriptions of PFA-induced coronary vasospasm were predominantly angiographic, characterizing the response as acute, transient, and voltage-dependent.^10–17^ Mechanistically, these observations appeared to parallel findings from non-coronary tissues; however, the interpretation was largely extrapolative, inferred from electroporation biology rather than directly demonstrated in the coronary circulation. From a physiological standpoint, beyond neurally mediated vasoconstriction and direct smooth-muscle contraction, the vascular endothelium likely plays a central role. High-intensity electric fields may induce transient endothelial poration, thereby altering local paracrine signalling. A shift towards a pro-vasoconstrictive milieu, marked by reduced nitric-oxide bioavailability or increased endothelin-1 release, could further amplify acute vascular hyperreactivity.^18^ Additionally, electroporation-related injury to circulating blood elements has been proposed as a complementary mechanism: local haemolysis may scavenge nitric oxide, impairing endothelial-dependent vasodilation, and release potent vasoconstrictive mediators.^19^

Recently, an integrated angiographic and histological characterization of the acute coronary response was provided.^20^ The study demonstrated that acute coronary vasospasm is peak-voltage dependent rather than energy-dose dependent, shows a brief delay to maximal constriction (∼5 min), and is associated with endothelial disruption and luminal protrusions. Furthermore, computational simulations suggest a clear relationship of distance from the electrode generating the electric field to the coronary artery, consistent with other preclinical observations.^21^ Together, these findings confirm that the coronary vasculature undergoes electroporation-linked processes analogous to those described in other tissues, while providing coronary-specific structural correlates and thresholds.

Delayed coronary vasospasm has been reported in isolated cases,^22,23^ occasionally with significant haemodynamic compromise.^24^ However, the mechanisms remain unknown. Autonomic imbalance,^25^ endothelial dysfunction, and haemolysis-related nitric-oxide depletion have been proposed, but no causal pathway has been demonstrated, and current evidence is insufficient to move beyond speculation.

Overall, PFA-induced coronary vasospasm should be understood as a specific manifestation of a broader and well-documented vascular reaction to electroporation, with coronary-specific evidence only now beginning to emerge—substantially strengthened by recent findings.

### Acute coronary artery spasm: clinical evidence

The first clinical report of a coronary artery spasm associated with PFA was published in 2021 during ablation at the mitral isthmus,^26^ challenging the concept of strict myocardial tissue selectivity of PFA. Since then, with an increasing number of PFA procedures and more extensive lesion delivery, additional instances of coronary vasospasms have been observed, underscoring their potential clinical relevance.

Current clinical evidence indicates that PFA-associated coronary artery spasm occurs in two distinct patterns. First, *proximity-related spasm* may occur when ablation is performed within the atrioventricular groove, most commonly at the cavotricuspid or mitral isthmus, where the electrical field lies in close spatial relationship to the right or circumflex coronary artery.^4,10,26,27^ Proximity-related coronary vasospasm has also been reported in 31% of patients during focal ventricular PFA using a solid-tip catheter.^28^

Second, *diffuse or generalized coronary spasm* may occur following ablation at sites remote from coronary arteries.^22,27,29^ Both entities may present either immediately, during energy delivery, or after a temporal delay. Delayed spasm has been reported with a median onset of 16–20 min after the last PFA application (interquartile range, 4–238 min and 4–315 minutes^24^), indicating that clinically relevant events may occur beyond the procedure completion.^24^

The electric field during PFA appears particularly prone to inducing vasospasm, which has been observed in 100% of cases when ablation is performed in direct proximity to a coronary artery, such as during CTI ablation.^10^ Importantly, most of these events remain subclinical, without accompanying electrocardiographic changes.^10,17^ Clinically manifest coronary spasm most commonly presents with ST-segment elevation and, less frequently, may progress to malignant ventricular arrhythmias, haemodynamic compromise, or cardiac arrest.^10,24,30^ ST-segment elevations are most commonly observed in the inferior leads, identified in 77% of reported cases.^30^

Consequently, the true incidence of coronary spasm is likely underestimated, as most reports derive from isolated case reports and subclinical spasms are not detected in clinical routine. In the MANIFEST-17K and MANIFEST-US surveys, including 17 642 and 41 968 patients undergoing PFA for atrial fibrillation, the reported incidence of coronary artery spasm was 0.14% and 0.10%, respectively.^4,27^ In MANIFEST-US, proximity-related events accounted for 0.07%, whereas remote or diffuse spasms occurred in 0.024% (10 of 41 968 procedures), confirming proximity-related spasm as the more common presentation.^27^ Consistent with prior observations, most vasospasms occurred during the procedure, were characterized by ST-segment elevation on the ECG, and lasted between 2 and 30 min.^27^ While these large registries provide important real-world insights into coronary safety, an important caveat is that the findings primarily reflect the PFA technologies and workflows represented in these studies and likely do not apply uniformly across different PFA platforms.

To date, at least 17 cases of delayed coronary artery spasm have been reported in the literature.^22,24,27,29,31–33^ The overall incidence of rare, life-threatening adverse events, including delayed myocardial ischaemia, malignant arrhythmias, and sudden cardiac death, has been reported as 0.16% (11 of 6721 PFA procedures).^24^

### Subacute and chronic structural changes of the coronary arteries

Application of high voltage electric fields with PFA near coronary arteries typically leads to spasm.^11,12,26^ The induced vasoconstriction resolves spontaneously over 25–30 min and was initially thought to be a transient event.^12^ However, chronic studies in animal models, although not identifying angiographically visible stenoses, have demonstrated histological injury at 4–8 weeks, characterized by neointimal hyperplasia and tunica media fibrosis (*Figure 1*).^11,12^ Whether these changes would manifest in human coronary arteries was initially unclear. Malyshev *et al*. performed coronary angiography a median of 11 months after ablation in 30 patients who had developed coronary artery spasm during PFA, and none had visible angiographic narrowing at the site of prior spasm.^34^ However, Tam and colleagues performed optical coherence tomography (OCT) acutely and at 3 months after PFA of the right atrial CTI using the pentaspline catheter in 19 patients.^16^ The authors found a mean coronary artery luminal narrowing at 3 months of 10.1%. At 19 months follow-up, it was noted that while there was reduction of coronary luminal area initially, there was no lesion progression.^35^ Importantly, although OCT demonstrated mild luminal reduction, no progression of stenosis or associated clinical ischaemic events have been reported to date, suggesting that the clinical significance of these structural findings remains uncertain. Pre-treatment with high dose intracoronary or intravenous nitroglycerin has been shown to mitigate the degree of acute spasm^10,17,36^; however, it is unclear whether this has any effect on the histologic changes which are likely due to direct injury from PFA.

**Figure 1:**
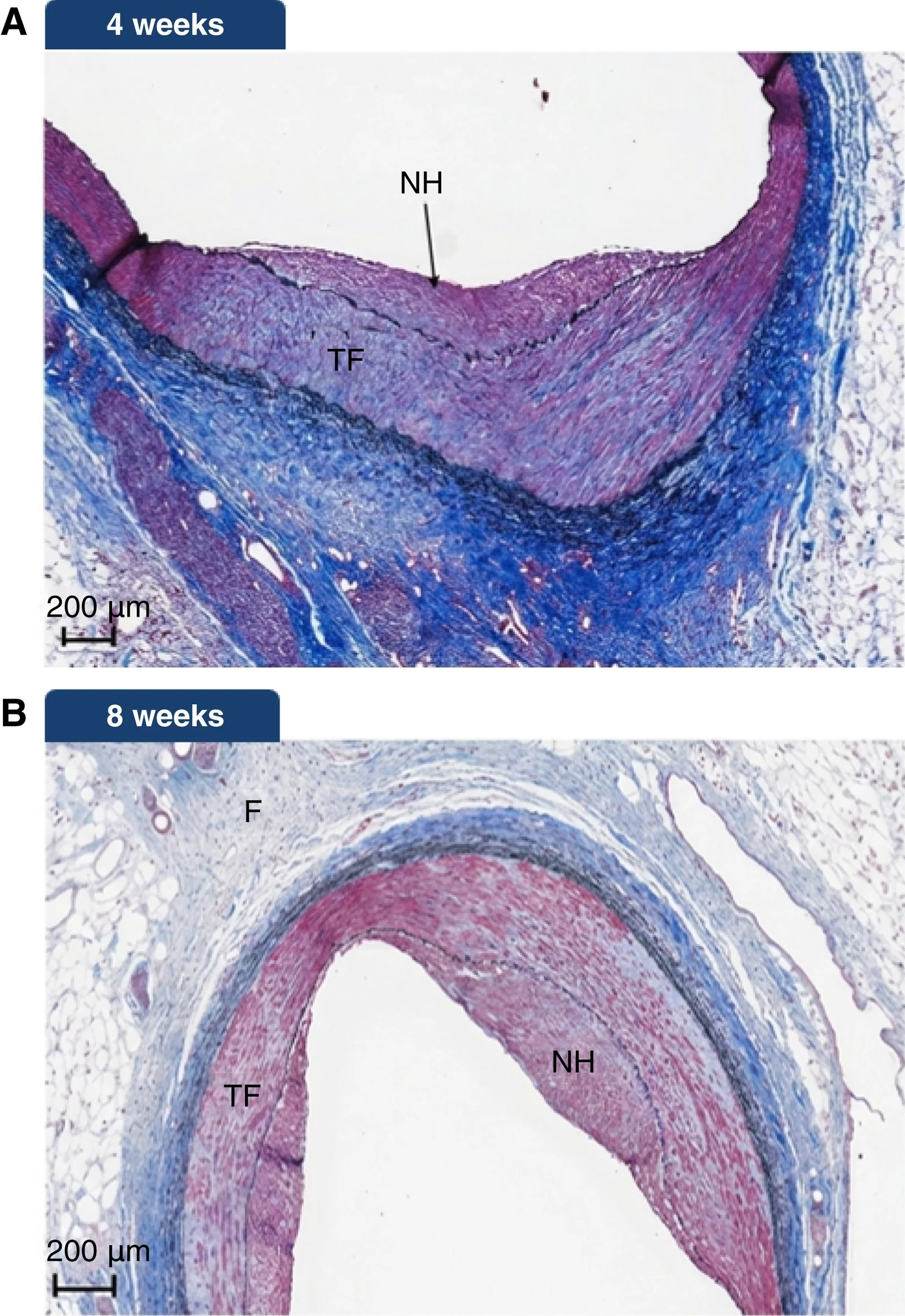
Haematoxylin and eosin stained histologic sections of the left anterior descending coronary artery 4 (*A*) and 8 weeks (*B*) after pulsed field ablation applied epicardially over the left anterior descending coronary artery using a linear catheter demonstrated mild neointimal hyperplasia (NH) and tunica media fibrosis (TF). F, adipose tissue fibrosis. Adapted from Higuchi *et al*.^12^ with permission.

Radiofrequency energy has been used for >30 years for CTI ablation with only rare cases of coronary artery injury.^37^ In preclinical studies comparing the effect of PFA to RF energy, the same histologic changes (predominantly neointimal hyperplasia) were also noted with RFA,^38^ with one study finding a greater degree of injury with RFA.^39^ Therefore, a mild degree of coronary artery injury is likely not unique to PFA. The neointimal hyperplasia after PFA was also found to occur regardless of energy vector, i.e. whether bipolar or unipolar PFA was applied.^38^ Whether alternate PFA waveforms (nanosecond, shorter pulse duration) would reduce the effects on coronary artery injury remains to be investigated. Historically, it was recommended with RF ablation that energy not be applied within 5 mm of a coronary artery. However, for PFA, it is not possible to provide a universal safety distance, as the extent of coronary exposure is likely influenced by waveform characteristics, catheter design, electrode configuration, lesion set, energy delivery parameters, and patient-specific factors. Accordingly, reported distance thresholds should be interpreted as platform-specific observations. For example, in a preclinical study using an 8F linear bipolar PFA catheter, coronary artery effects were observed when energy was delivered within 6.5 mm of the vessel wall.^21^ However, when it cannot be avoided, pre-treatment with high dose (2–3 mg) IV nitroglycerin (NTG) should be utilized. While preclinical data suggest that the effect may be peak energy-dependent rather than cumulative,^40^ operators should still aim to avoid unnecessary PFA applications and limit exposure to the minimum required for procedural efficacy. In at least one case, application of PFA to a right coronary artery led to acute plaque rupture and myocardial infarction.^24^ Therefore, knowledge of a patient’s coronary anatomy and whether lesions exist in collateral vessels are also important to minimize complications. A critical unresolved question is whether these histological changes are clinically silent epiphenomena or modifiers of long-term coronary risk, particularly in patients with pre-existing coronary artery disease or endothelial dysfunction.

### Clinical implications, management, and monitoring

The occurrence of coronary vasospasm during ablation procedures carries important clinical implications, requiring targeted management and vigilant patient monitoring. Management strategies should be tailored according to the specific coronary safety phenotype, as preventive measures for proximity-related spasm may not be applicable to diffuse vasospasm or subacute coronary remodelling. First, when ablating within the atrioventricular groove, such as during CTI or mitral isthmus ablation, preventive strategies to reduce proximity-related coronary vasospasm should be considered. These include careful selection of the energy source. In situations where ablation must be performed in close proximity to a major epicardial coronary artery, thermal ablation (e.g. using RF or dual-energy catheters) may be considered until more robust platform-specific safety data become available. Alternatively, if PFA is used, prophylactic nitroglycerin may be administered via intravenous, intra-atrial, or intracoronary routes prior to energy delivery to reduce the risk of coronary vasospasm.^41^

Accumulating evidence suggests that coronary spasm can be effectively prevented by prophylactic NTG administration. For example, in the prospective multicentre ADVANTAGE Phase II study, a standardized nitroglycerin protocol was recommended to prevent coronary spasm during CTI ablation with a focal PFA catheter, consisting of a 3-mg intravenous bolus administered 1 min before the first PFA application, followed by additional 2-mg doses every 2 min until completion of the lesion set.^42^ Concomitant administration of intravenous vasopressors, such as phenylephrine, was used to prevent hypotension. Using this approach, CTI ablation with a focal short-linear PFA catheter was performed in 141 patients, with successful implementation of the prophylactic nitroglycerin protocol in 99.3% of cases and no observed ST-segment changes or ventricular fibrillation. The mean nitroglycerin dose was 4 ± 2 mg per patient, and more than half of patients received only the initial 3-mg bolus.

Despite these encouraging data, coronary spasm may still occur despite nitrate administration,^30^ underscoring the importance of appropriate dosing, timing, and awareness of nitroglycerin’s short half-life, which often necessitates repeated additional doses every few minutes.^17^ In contrast to proximity-related events, diffuse or remote coronary spasms remain unpredictable, and patient-specific risk factors are not yet clearly defined.^24^ Certain clinical characteristics, including pre-existing coronary artery disease, prior Prinzmetal’s angina, heart failure, or specific ethnic backgrounds, may have an increased susceptibility, but this requires further mechanistic investigation.^24,27,30^ Furthermore, the presence of these isolated reports could potentially be an indicator of autonomic or endothelial mechanisms that contribute to longer-term coronary vulnerability following PFA. Future work is needed to understand the long-term vascular consequences of PFA and how that compares to the RF standard of care.

### Clinical features of coronary artery spasm

Clinically, coronary spasm may manifest along a broad severity spectrum, ranging from transient ST-segment elevation to profound haemodynamic compromise or cardiogenic shock, particularly in delayed or diffuse presentations.^17^ Patients with a history of spasm should not have nitrates or calcium channel blockers held prior to the ablation procedure. Electrophysiology teams should be prepared for rapid escalation of care during the ablation procedure, including emergent coronary angiography for intracoronary nitrate administration, percutaneous coronary intervention in the setting of plaque rupture, or, in rare cases, temporary mechanical circulatory support. Although most reported events occurred during post-procedural recovery, delayed spasm up to 6 h and sudden cardiac death within the first month after PFA have been described. At present, no evidence-based recommendations exist regarding the optimal duration of post-procedural monitoring or the role of routine oral nitrate therapy after PFA. Although these events remain uncommon, recent reports of delayed coronary vasospasm, myocardial ischaemia, and sudden cardiac death highlight important gaps in our understanding of the coronary effects of PFA and support continued vigilance, particularly as the technology is increasingly applied to complex non-PVI lesion sets (*Figure 2*).

**Figure 2:**
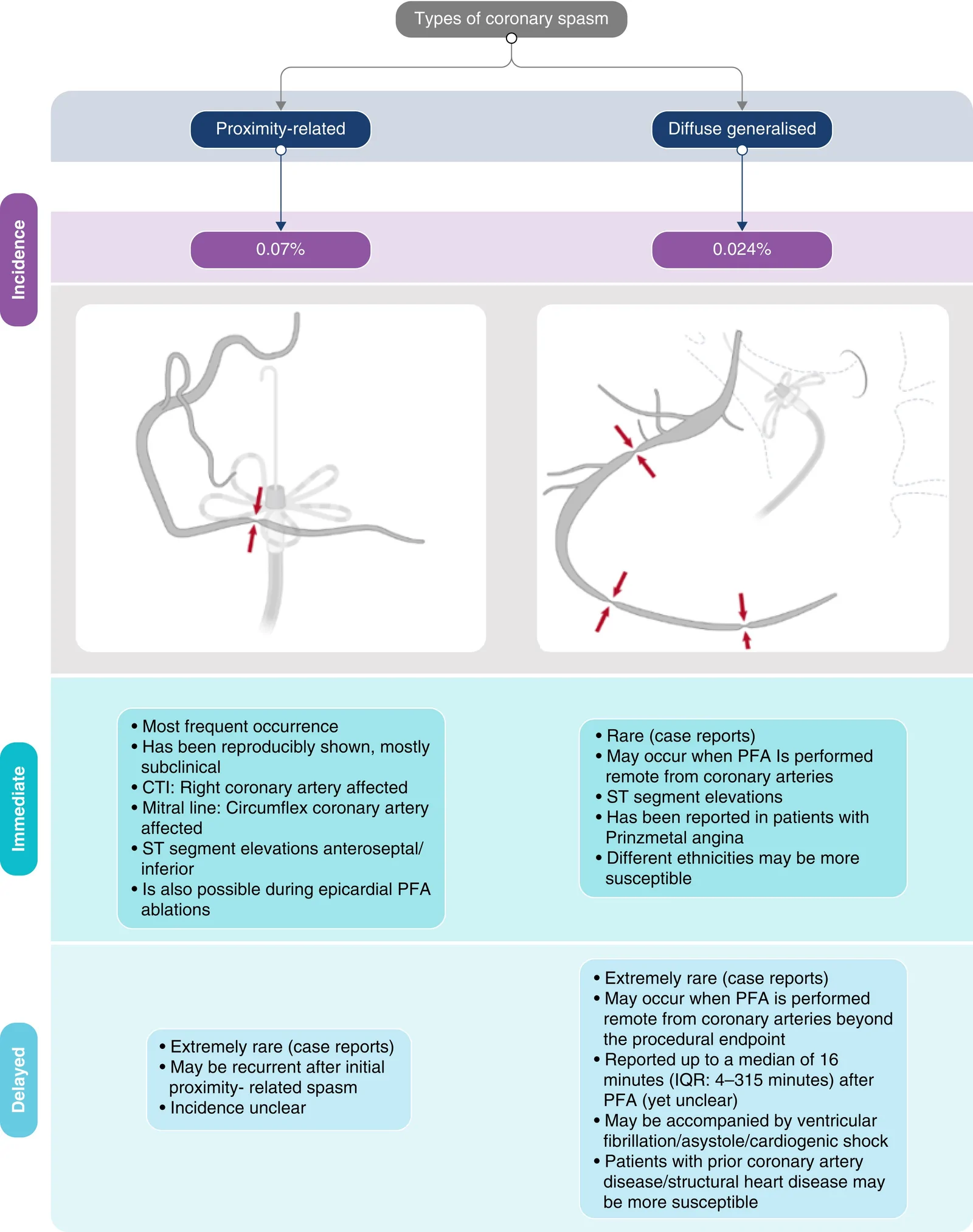
Types, incidence, timing, and clinical spectrum of PFA-associated coronary vasospasm. Proximity-related spasm (≈0.07%) occurs near the ablation site and is the most common pattern, whereas diffuse/generalized spasm (≈0.024%) affects multiple coronary segments remotely. Events may present immediately (more frequent) or in a delayed fashion (rare), with potential ST-segment elevation and severe arrhythmic or haemodynamic complications.

## Haemolysis

### Mechanisms

Haemolysis during PFA results from IRE of circulating RBCs exposed to intense electric fields. The electric pulses transiently disrupt cell membranes, inducing osmotic imbalance, cellular swelling, and membrane rupture, with subsequent release of free haemoglobin (fHb) and cytosolic enzymes such as lactate dehydrogenase (LDH) (*Figure 3*). Unlike thermal or mechanical haemolysis, this injury is likely non-thermal and field-dependent, occurring without tissue heating or shear stress.^50^ From a biophysical standpoint, the small dimensions of RBCs would be expected to result in a higher electroporation threshold than that of larger cells such as cardiomyocytes. This arises from the size dependence of the induced transmembrane potential, which scales with cell radius.^43^ However, in the near vicinity of any PFA catheter, the electric field intensity is high enough to induce electroporation of RBCs.

**Figure 3:**
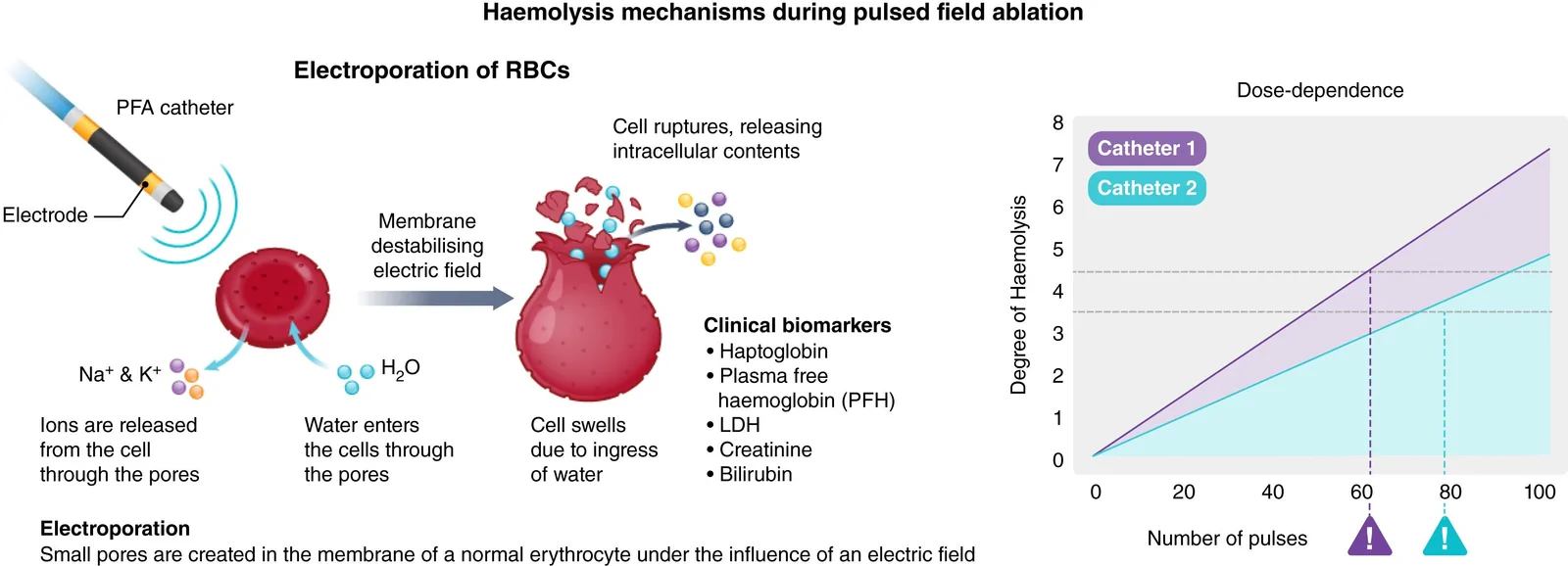
Mechanisms of haemolysis during pulsed field ablation. High-voltage electric pulses cause electroporation of red blood cell membranes, leading to osmotic imbalance, swelling, and rupture with release of free haemoglobin (fHb) and lactate dehydrogenase (LDH). The severity of haemolysis increases proportionally with the number of PFA applications and voltage amplitude.

For a given PFA platform, the extent of RBC injury increases with pulse amplitude, number of applications, and total electrode exposure time. Both clinical and experimental data demonstrate a clear dose-dependent relationship.^44,45^ For example, for the pentaspline catheter, significant haemolysis has been typically observed beyond 50–70 applications with higher risk for AKI when exceeding 100 applications. However, because of the marked differences across PFA systems (*Figure 4*), it is not possible to provide a general maximum number of applications to limit the risk of significant haemolysis or AKI. These thresholds are likely to vary substantially across platforms.^44,45^ Additionally, catheter geometry also modulates haemolytic intensity: multi-spline ‘single-shot’ systems generate greater elevations in haemolysis biomarkers compared with focal or balloon-in-basket devices, where the electrodes are relatively insulated from the blood pool.^48^ Recent prospective comparative data demonstrated significant differences in laboratory-defined severe intravascular haemolysis across commercially available PFA technologies, occurring in 37.0% of patients treated with pentaspline catheter, 26.1% with circular-array catheter, and 14.7% with a lattice tip catheter, confirming catheter design as an important determinant of haemolytic burden despite the absence of clinically significant renal impairment.^49^ Finally, poor electrode-tissue contact has also been associated with a greater degree of haemolysis.^48,50^

**Figure 4:**
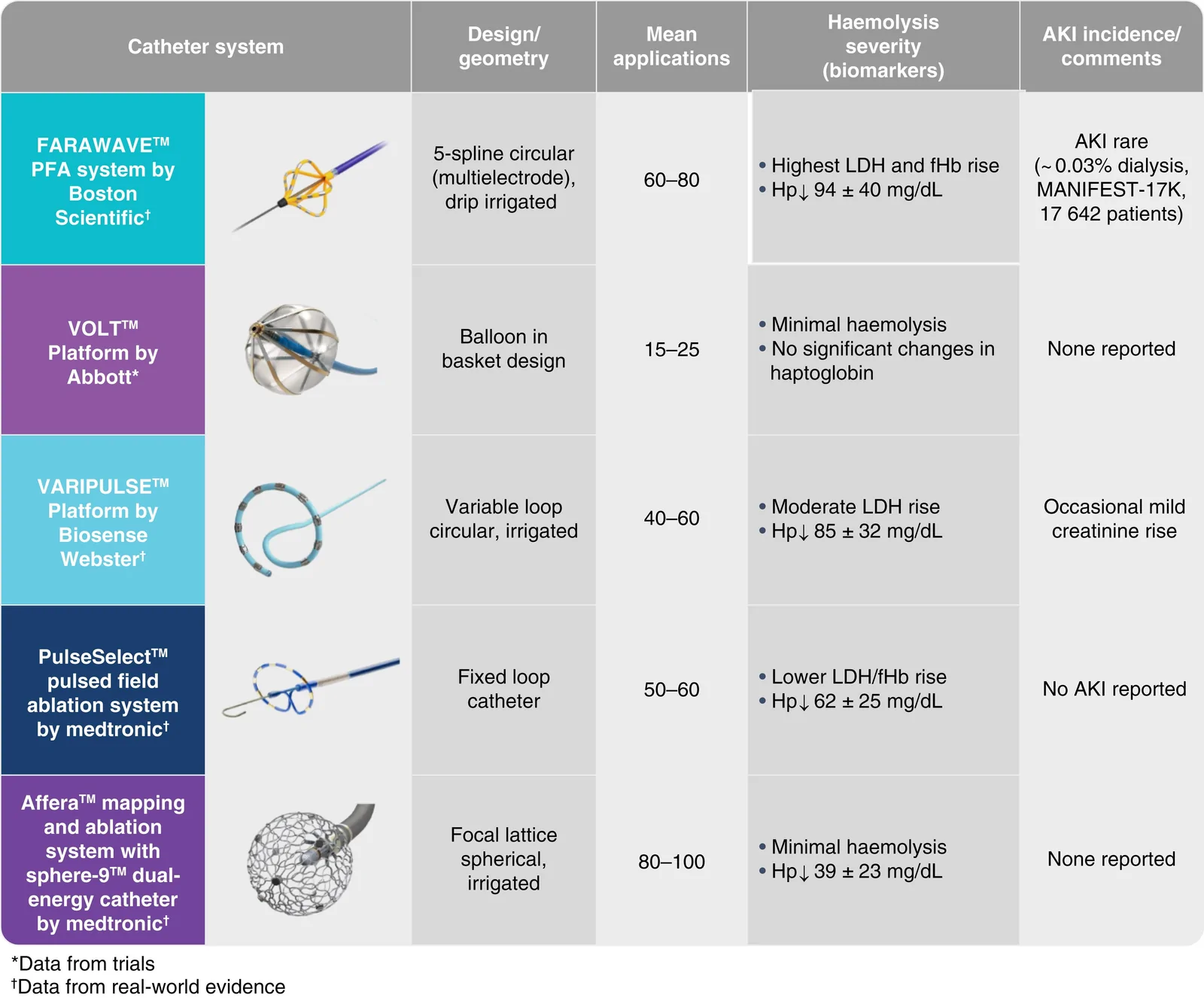
Comparison of haemolysis across PFA systems. Comparative analysis of haemolysis severity and renal risk across major PFA catheter systems. Data summarized from multicentre clinical and preclinical studies.^4,44–47^ AKI, acute kidney injury; Hp, haptoglobin; LDH, lactose dehydrogenase; Hb, haemoglobin; fHb, free haemoglobin.

Thus, PFA-related haemolysis represents a predictable electroporation effect inherent to the technology, whose extent depends on the interplay between energy dose, catheter–blood interface, electrode-tissue contact, catheter ablation footprint, and device design.

### Biomarkers

Haemolysis after PFA is characterized by a consistent biochemical triad: elevation of LDH, increase in plasma free haemoglobin, and reduction in haptoglobin. Lactate dehydrogenase levels rise rapidly after ablation and correlate with both the number of pulses delivered and total energy applied.^44,51^ As LDH is a universal enzyme found in all cell types, its elevation is not highly specific for haemolysis and may also be linked to cardiac tissue damage. Free haemoglobin is the most specific acute marker of intravascular RBC rupture, increasing three- to five-fold after PFA in proportion to pulse count.^46,48^

Haptoglobin levels decrease in parallel, often below 0.04 g/L, reflecting haemoglobin binding and scavenging.^45,52^ Ancillary markers such as indirect bilirubin or urinary haemoglobin are less specific but can support the diagnosis. Adequate pre-ablation hydration markedly reduces biomarker elevations and the risk of AKI.^53^

Altogether, the pattern of LDH↑/fHb↑/Hp↓ constitutes the biochemical fingerprint of PFA-related haemolysis and provides a practical framework for its detection and procedural optimization. Consequently, post-procedure monitoring of LDH, free haemoglobin, and haptoglobin could be considered when haemoglobinuria or AKI is suspected. Pre-procedural biomarker monitoring strategies could include baseline renal function in all patients to establish potential high-risk scenarios and post-procedure creatinine and urinalysis could be considered in high-risk cases.

### Clinical evidence

Biochemical haemolysis occurs in the vast majority of PFA procedures, irrespective of catheter design or energy settings. Across multicentre studies, LDH and plasma fHb increase in more than 90% of cases, confirming that transient exposure of circulating blood to pulsed electric fields consistently induces RBC electroporation and rupture.^44,51^

Despite this near-universal biochemical phenomenon, clinically significant consequences are rare. Transient haemoglobinuria occurs in 10–20% of procedures, typically resolving within 24–48 h. Severe haemolysis-related AKI has been described only in isolated reports, generally after extensive energy delivery and may be mitigated by more aggressive periprocedural hydration.^44–46^

In the MANIFEST-17K registry encompassing 17 642 patients treated with the pentaspline PFA catheter, five cases (≈0.03%) of AKI requiring temporary haemodialysis were reported, all occurring after a very high number of pulse applications and resolving completely.^4^ As these data were generated using a specific PFA platform, the rates of haemodialysis cannot be extrapolated to other catheter technologies or waveforms. In addition, special caution is warranted when combining different PFA catheters. For instance, using both a pentaspline catheter and a focal quadripolar PFA catheter in the same patient, since the delivered doses are cumulative, increases the total energy exposure and raises the risk of haemolysis-related renal impairment.

Overall, clinical evidence suggests that PFA-associated haemolysis is ubiquitous but predominantly subclinical, with renal injury confined to rare, high-dose, or poorly hydrated patients.

### Mitigation strategies

Although biochemical haemolysis is universal after PFA, its clinical impact can be effectively minimized through pre-procedural procedural and periprocedural optimization. Definition of high-risk scenarios should help adopt preventive measures to mitigate associated risks. In this sense, patients with pre-existing renal dysfunction, heart failure limiting intra-procedural or post-procedural hydration, or baseline haematological abnormalities may be particularly vulnerable to the downstream consequences of haemolysis and therefore warrant increased clinical vigilance.^44,54^ Consistent with this, available clinical reports suggest that clinically significant AKI occurs predominantly in the setting of extensive energy delivery.^44^ This may occur when involving extensive posterior wall or extra-PV ablation or the combined use of more than one PFA catheter. Increased risks could also be associated with poor electrode-tissue contact. As discussed above, experimental and clinical data confirm that haemolysis is dose- and contact-dependent, increasing with higher pulse counts, excessive blood exposure, or poor electrode–tissue coupling.^48,55^

Consequently, maintaining adequate catheter contact reduces intravascular exposure, whereas minimizing number of applications and avoiding unnecessary overlapping lesions are key preventive measures. At present, many PFA catheters lack integrated contact force sensing; however, impedance-based contact assessment is now available in many single-shot and large focal devices. Use of intracardiac echocardiography, to assure electrode-tissue contact prior to ablation, may also play a role in reducing haemolysis.^45,56^

Periprocedural hydration remains the cornerstone of renal protection. In a multicentre series, Mohanty *et al*. showed that administering ≥2 L of isotonic saline virtually eliminated post-PFA haemoglobinuria and prevented haemolysis-related AKI.^53^ Experimental data from Yuan *et al*. further suggest that gadolinium-diethylenetriamine-penta-acetic acid (Gd-DTPA) may attenuate erythrocyte electroporation by stiffening cell membranes, reducing haemolysis both *in vitro* and *in vivo*.^57^

Together, avoidance of excessive lesion delivery, optimized catheter-tissue contact, catheter-specific hemolytic profile awareness, proactive hydration with saline, and potentially biochemical membrane stabilization represent complementary strategies to prevent clinically significant PFA-induced haemolysis and renal complications.

## Silent cerebral events

### Mechanisms

Left atrial ablation historically carries a risk of cerebral embolic events. While clinically apparent strokes are rare (<1%), MRI studies reveal a much higher incidence of silent cerebral emboli or lesions (SCE/SCL) in up to 14% of RF ablations^58^ and around 9% in PFA trials.^59–62^ Interpretation of reported SCL rates requires caution, as incidence is highly dependent on study methodology including MRI timing, use of baseline MRI, magnetic field strength, diffusion-weighted imaging protocols, anticoagulation strategy, extent of ablation, catheter exchanges, sheath management, and procedural workflow. Recent registry data confirm MRI evidence of asymptomatic cerebral emboli following PFA, indicating that although catastrophic complications like AEF are avoided, embolic risk may persist.

The origins of SCE/SCL following ablation remain unclear, partly because imaging cannot distinguish between solid and gaseous emboli. Microbubble generation has been observed both anecdotally and during PFA clinical trials and results from electrolysis and gas formation caused by high-amplitude current delivery to blood.^63^ Radiofrequency ablation can also produce bubbles through localized overheating.^64^ In contrast, while solid particles from RF ablation are known to arise from coagulum deposition, it remains uncertain whether PFA may also generate solid particulates. As such, pinpointing the mechanism (either solid or gaseous) of SCE/SCL remains difficult.

In addition to intra-procedural mechanisms, delayed embolic phenomena may also contribute. Atrial stunning following PFA, characterized by transient mechanical dysfunction and reduced contractility,^65^ may promote blood stasis and thrombus formation, thereby representing a potential mechanism for delayed cerebral embolism. Thus, more extensive ablation than necessary should be avoided.

Importantly, it cannot be conclusively stated that the observed rates of SCLs and stroke are solely energy related. Air ingress during catheter or sheath manipulation remains a shared, non-energy related procedural concern across ablation modalities.

### Risk factors

Several clinical and procedural factors can significantly influence embolic susceptibility, as previously reviewed.^66^ In alignment with air ingress during sheath manipulation, numerous catheter exchanges have been shown to increase the likelihood of silent cerebral infarcts.^67^ Suboptimal periprocedural anticoagulation is widely recognized as a major predictor of SCE/SCL formation^68^ due to inadequate anticoagulation permitting thrombus formation on foreign surfaces, such as the catheter or sheath. Takami *et al*. demonstrated that microembolic signals occur predominantly during PFA energy delivery using the pentaspline catheter, particularly in the basket configuration and in the presence of overcurrent errors (303 errors), highlighting the importance of catheter configuration and procedural workflow as potential determinants of cerebral embolic risk when using the pentaspline catheter. Overall choice of PFA catheter may influence SCE/SCL occurrence; however, comparisons across technologies should be interpreted cautiously because reported event rates are substantially influenced by a variety of factors including imaging protocols, procedural workflow, anticoagulation strategies, and overall study design.^69–72^

### Clinical implications

Recent evidence has produced mixed findings regarding the relative cerebral safety of PFA. In the neurological substudy of the randomized ADVENT trial, MRI-detected SCE/SCL rates were low and did not suggest an excess neurological risk with PFA compared with thermal ablation.^73^ Similarly, a recent systematic review and network meta-analysis found no significant difference in SCE incidence between PFA and thermal ablation, although substantial variability was observed across catheter technologies and studies.^71^ In contrast, emerging real-world data, including findings from the EMBOL-AF registry,^74^ suggest a potentially higher embolic burden with PFA than initially anticipated. Supporting this possibility, a pilot randomized MRI study reported a numerically higher incidence of SCL following PFA than RF ablation (12.9% vs. 6.5%), although the difference was not statistically significant.^75^ Taken together, these findings highlight that cerebral embolic risk remains incompletely understood and likely depends as much on procedural workflow, imaging methodology, and catheter design as on the energy source itself.

Despite increasing recognition of MRI-detected silent cerebral events after AF ablation, their clinical and neurocognitive significance remains incompletely understood. Most events are asymptomatic and only detectable through advanced imaging such as diffusion-weighted MRI.^66^ Intra-procedural monitoring tools such as transcranial Doppler can identify microembolic signals, but again their clinical relevance is unclear. Prevention relies on strict anticoagulation protocols, minimal catheter exchanges, and careful sheath management. Notably, emerging evidence shows that PFA catheter design influences microembolic burden, underscoring the potential importance of PFA catheter selection.

## Pulsed field ablation interactions with implantable devices

As AF ablation expands into older and more comorbid populations, coexistence of PFA technology with cardiac implantable electronic devices (CIEDs) has become increasingly common. Unlike thermal ablation modalities, PFA delivers high-amplitude pulsed electric fields that can interact with nearby metallic structures or highly conductive parts, including CIEDs and left atrial appendage occlusion (LAAO) devices.

### Mechanisms

The risk and severity of these interactions are likely influenced by the specific PFA technology used and the type of implanted device. Differences in catheter design, including monopolar vs. bipolar configurations, affect the spatial distribution of the electric field and therefore govern the possible interactions of PFA with CIEDs. Monopolar systems, in which the electric current flows from the ablation electrode on the catheter to a return patch on the back of the patient, generate a broader electric field distribution and may theoretically confer a higher risk of interaction due to greater field dispersion. However, the highest electric field intensities are still concentrated around the catheter in monopolar systems, and it remains uncertain at what point the electric field intensity is sufficient to induce permanent device damage, or if sustained electric field is the critical component.

Waveform characteristics also play a critical role. Pulse parameters, including duration and pulse delay, will define the spectral frequency content of the PFA signal affecting the electromagnetic interference profile. These factors may affect coupling with device leads and internal circuitry, potentially triggering unintended sensing or device responses. Additionally, the amplitude (voltage or current) and workflow-specific factors may further modulate risk. Accordingly, the interaction profile between PFA and CIEDs is highly system-dependent and platform-dependent.

### Cardiac implantable electronic device

Pulsed field ablation has demonstrated both transient and, rarely, permanent interactions with CIEDs. Case reports have described irreversible intracardiac defibrillator (ICD) damage when monopolar PFA was delivered in presumed contact with the SVC coil.^76^ Recently, another case of permanent damage to an ICD was reported using the same monopolar PFA system.^77^ In contrast to this evidence, controlled preclinical data from an *ex vivo* assessment [44 devices (16 pacemaker, 21 ICDs, 7 CRT-P/D), 1980 PFA applications] demonstrated no change in device settings, functionality, and no macroscopic damage to the devices.^78^ Notably, differences in catheter technology likely explain these divergent findings: the reported cases of permanent damage involved a large focal monopolar system, whereas the preclinical study used a bipolar pentaspline catheter. Besides permanent damage to CIEDs, which seems to be extremely rare, PFA can also cause electromagnetic interference changing sensing behaviour of CIEDs and lead–catheter interactions. Potential hazards include CIED oversensing of PFA signals, stunning or damage to tissue surrounding a CIED,^79^ induction of an arrhythmia,^80^ arcing, or device dislodgement.

Clinical data using bipolar systems further support a favourable safety profile. In the DISRUPT-AF registry (*n* = 394 with CIEDs), no device-related complications or increase in acute adverse events were observed.^81^ However, interpretation of these findings is limited by the lack of detailed information regarding catheter-lead proximity during energy delivery, which is likely a key determinant of risk. Overall, these data suggest that contemporary bipolar PFA systems are generally safe in routine workflows, although caution remains warranted in scenarios involving close proximity to device components. This may become more prevalent as PFA is increasingly used for ablation in the right ventricle. Furthermore, as new technologies are released, distance-safety thresholds should be assessed, especially in monopolar systems.

From a practical standpoint, patients with CIEDs undergoing PFA should undergo appropriate pre- and post-procedural device assessment, particularly when energy delivery is anticipated near device components. Special attention should be given to patients who are pacemaker dependent, in whom temporary reprogramming strategies, such as asynchronous pacing, may be considered to minimize the risk of pacing inhibition during energy delivery. In patients with ICDs, temporary suspension of ICD therapies should be performed preprocedurally to prevent inappropriate device therapies. Continuous rhythm and haemodynamic monitoring during energy delivery may also be considered to detect transient device interactions that may not be evident on routine interrogation. Overall, when feasible, unnecessary energy delivery in close proximity to device leads or generators should be avoided until platform-specific safety thresholds are better defined and catheter-lead electrode contact should be altogether avoided.

### Left atrial appendage occlusion devices

Patients referred for AF ablation and LAAO devices frequently represent an overlapping cohort, creating scenarios in which PFA could be performed either prior to or following device implantation. In many concomitant cases (e.g. combined PFA and LAAC in one procedure), operators opt to perform PFA prior to LAAO deployment, in part to reduce the risk of device-field interactions. However, a substantial population of patients with pre-existing LAAO devices will subsequently undergo ablation, making this an increasingly relevant clinical consideration.

Emerging preclinical and clinical data suggest that PFA may interact electrically with LAAO devices. An *in vitro* study demonstrated that PFA delivered in close proximity (0–1 mm) to unendothelialized LAAO devices has the potential to cause arcing, observed across both monopolar and bipolar systems. At a separation distance of ∼3 mm, there interactions were no longer observed, suggesting that interactions may be less likely at greater separation distances. However, these findings are derived exclusively from an *in vitro* model and should not be interpreted as a validated clinical safety threshold.^82^

### Future perspectives

Several key questions remain unresolved. It is unclear which procedural variables are important to these hazards, including catheter lead distance, or even CIED device manufacturer. Additionally, the safety profile of PFA in patients with newer technologies, such as leadless pacemakers, remains incompletely defined. Expanded clinical registries and prospective studies are needed to better characterize real-world outcomes in patients with CIEDs undergoing PFA. These data will be particularly important as PFA indications expand to include regions such as the superior vena cava, where proximity to device leads may increase the likelihood of interaction.^83^

## Comparison to thermal technologies

The clinical adoption of PFA represents a paradigm shift away from conventional thermal modalities—RF and cryoballoon (CB) ablation—driven by a search for more selective and safer energy sources. While thermal technologies rely on conductive heating (>52°C) or freeze-thaw cycling to induce tissue necrosis, PFA utilizes non-thermal IRE as stated above to disrupt cardiomyocyte membrane integrity while largely sparing surrounding collagenous structures. Although neural membranes can be sensitive to electric fields, larger nerves are relatively protected by insulating factors such as the myelin sheath. This selectivity has profound implications for the incidence and management of procedural complications (*Figure 5*). However, as discussed throughout this review, PFA is associated with its own safety profile, including coronary vasospasm and haemolysis-related AKI. Therefore, contemporary comparisons between ablation technologies should consider both the reduction in thermal injury and the emergence of technology-specific adverse events. Moreover, a recent network meta-analysis of randomized trials found broadly comparable conventional safety and efficacy outcomes across RF, CB, laser balloon, PFA, and lattice-tip dual-energy ablation technologies, while identifying differences in procedural efficiency and AF-burden endpoints.^84^

**Figure 5:**
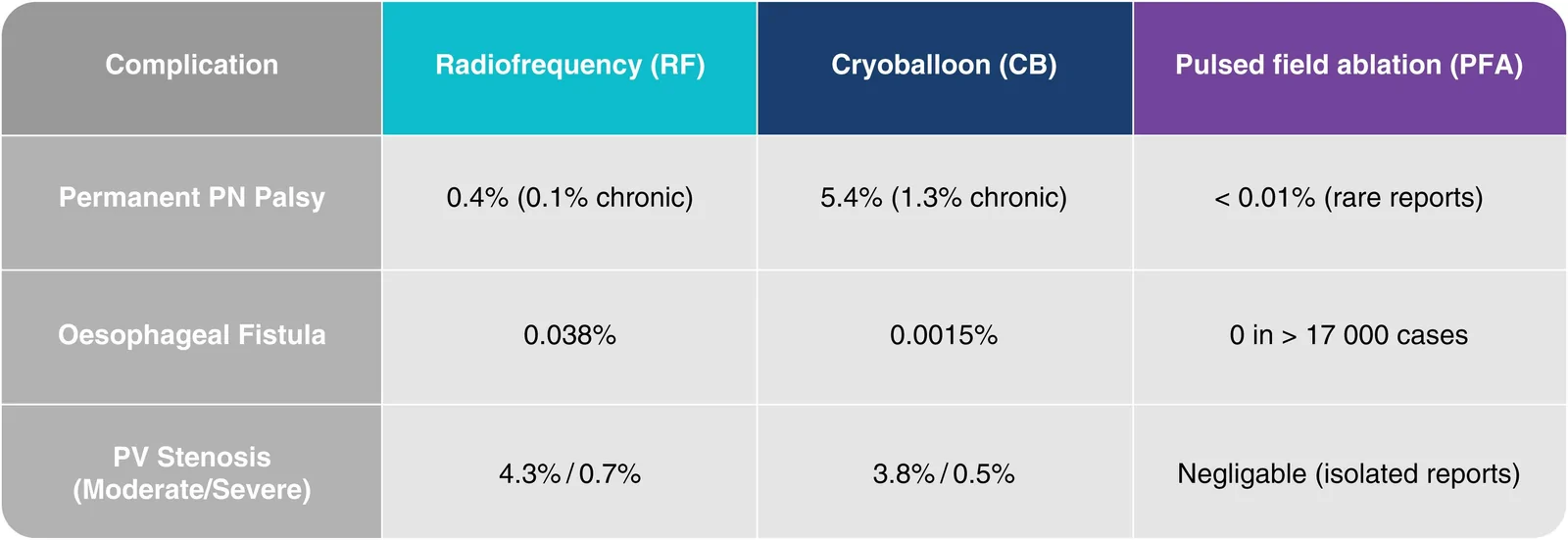
Comparative clinical safety of PFA vs. thermal ablation.

### Phrenic nerve injury

Phrenic nerve injury is a well-recognized complication of thermal AF ablation, particularly during CB procedures, where incidence rates of permanent PNI are ∼1.9%.^83^ Thermal injury involves axonal degeneration due to the nerve’s anatomical proximity to the right PVs.

In the PFA landscape, permanent PN injury appears exceptionally rare (*Figure 6*).^85^ The MANIFEST-17K study, involving over 17 000 patients, reported transient PNI in only 0.06% of cases and zero instances of permanent palsy.^4^ While thermal ablation requires continuous PN pacing and diaphragmatic monitoring, PFA-induced PN effects are typically limited to transient ‘stunning’ or capture during pulse delivery, with rapid functional recovery. However, recent series using sequential compound motor action potential (CMAP) monitoring have reported additional nuance, reporting acute PNI in 26 patients (40.6%).^86^ While the majority recovered, incomplete recovery was observed in 24% (6/25) at discharge, and persistent dysfunction was confirmed in one case at 3 months. Although these frequently observed acute CMAP-detected findings may reflect a higher sensitivity of monitoring, their clinical relevance remains uncertain, as this approach may detect subclinical or clinically irrelevant phrenic nerve impairment. However, isolated case reports of persistent palsy have also emerged recently,^87^ underscoring the need for further vigilance regarding this adverse event. These complications may be device-specific; certain PFA form factors may stretch the PV towards the nerve, mimicking the mechanical stress seen in CB ablation (*Figure 1*).

**Figure 6:**
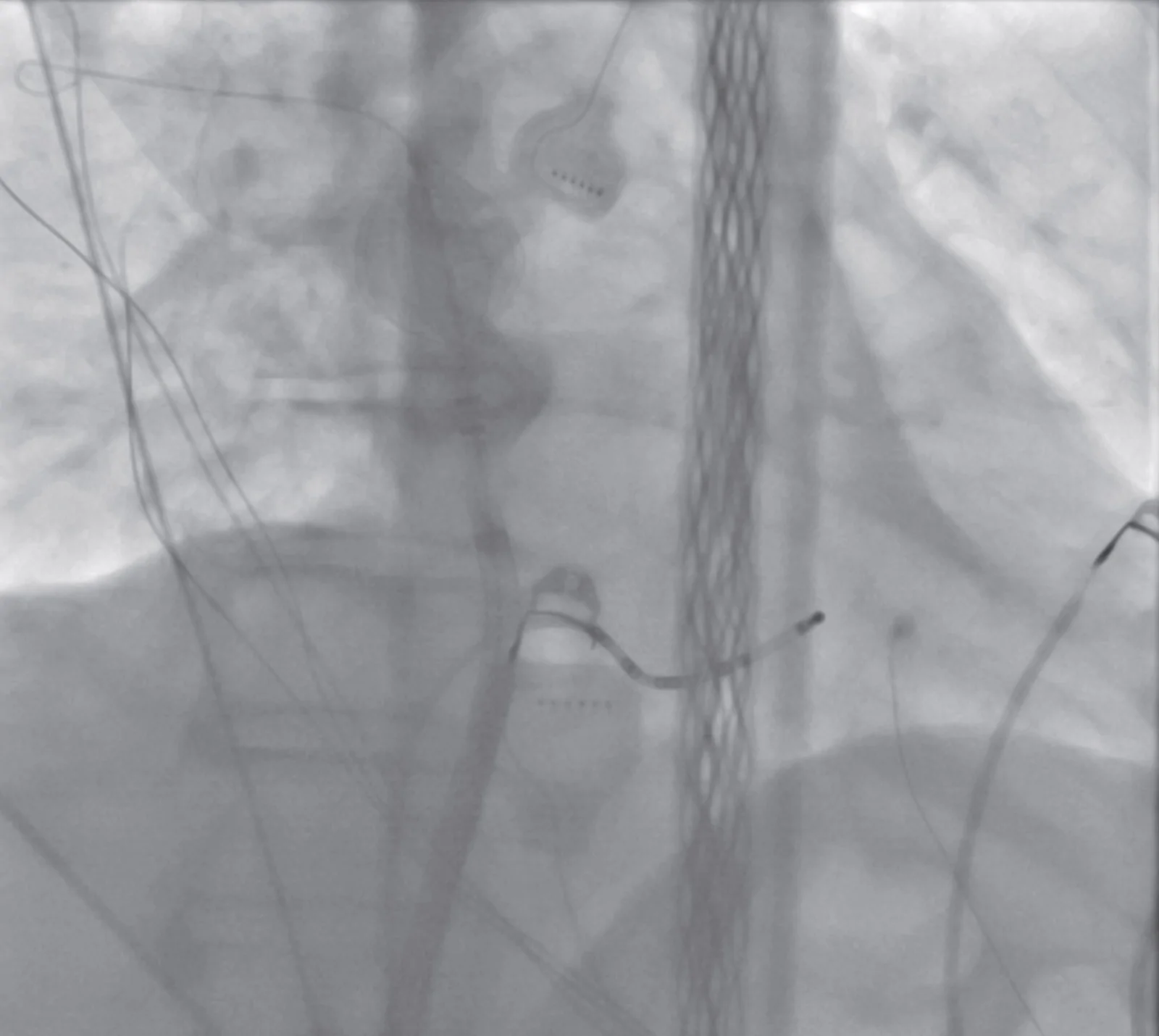
Right phrenic nerve stunning during PFA. Fluoroscopic image in the right anterior oblique (RAO) projection demonstrating an elevated right hemidiaphragm following pulsed field ablation (PFA) delivery in the right superior pulmonary vein (RSPV). A ‘balloon-in-basket’ PFA device and its corresponding guidewire are positioned within the RSPV. A decapolar reference catheter is visible in the coronary sinus (CS).

### Oesophageal effects

Oesophageal injury, specifically the rare but often fatal AEF, is the most feared complication of thermal ablation. Thermal energy causes indiscriminate protein denaturation and conductive damage to the oesophageal wall. Pulsed field ablation’s tissue-selective mechanism offers a significant advantage, as cardiomyocytes can have a lower electroporation threshold than oesophageal tissue and oesophageal tissue is relatively protected by attenuation of the electric field and any potential heating across the atrial-oesophageal interface.^88^

The RF and CB ablations are associated with an AEF incidence of ∼0.038% and 0.0015%, respectively (*P* < 0.0001).^89^ In contrast, no cases of AEF have been reported to date in more than 17 000 (and more recently estimated up to 500 000) procedures worldwide.^4^ This inherent safety allows for more confident ablation along the posterior left atrial wall without the intensive temperature monitoring or mechanical deviation required for thermal modalities.

### Pulmonary vein stenosis

Pulmonary vein stenosis (PVS) results from thermal injury to the vascular wall, causing collagen contraction and reactive intimal hyperplasia. In a large RF series, 31.4% of patients showed mild stenosis, 4.3% moderate, and 0.7% severe.^90^ Moderate PVS is also more frequent after laser balloon than CB ablation (8.2% vs. 3.8%; *P* = 0.03).^9^ The risk of PVS is particularly higher if high-power short-duration RF application is used (10.9% vs. 0.3% in conventional RF application, *P* < 0.001).^91^

Pulsed field ablation preserves the extracellular matrix and structural integrity of the vasculature. Clinical imaging follow-ups consistently show that PV narrowing after PFA is negligible and lacks clinical sequelae, allowing for effective isolation without the risk of long-term cicatricial narrowing.^92^ Nevertheless, clinicians should remain vigilant as isolated reports of PV stenosis after PFA have recently been described.^93^

### Troponin biomarkers

Myocardial biomarker release has historically served as a surrogate for the volume of tissue necrosis achieved. Pulsed field ablation procedures consistently lead to significantly higher peak levels of high-sensitivity troponin (hs-cTnT), myoglobin, and CK-MB compared to thermal ablation, often two to four times higher.^94^

In thermal ablation, massive troponin release may signal collateral damage. Interestingly, markers of systemic inflammation (CRP) remain comparable, although some studies show CB is associated with greater increases in leukocytes (Delta 2.5 vs. 1.1 times; *P* = 0.05) and CRP (Delta 5.8 vs. 3.4 mg/L; *P* = 0.02) than PFA, suggesting that the heightened myocardial injury in PFA does not translate into increased systemic stress. The overall safety profile of PFA and its principal mechanism-specific adverse effects are summarized in *Figure 7*.^95^

**Figure 7:**
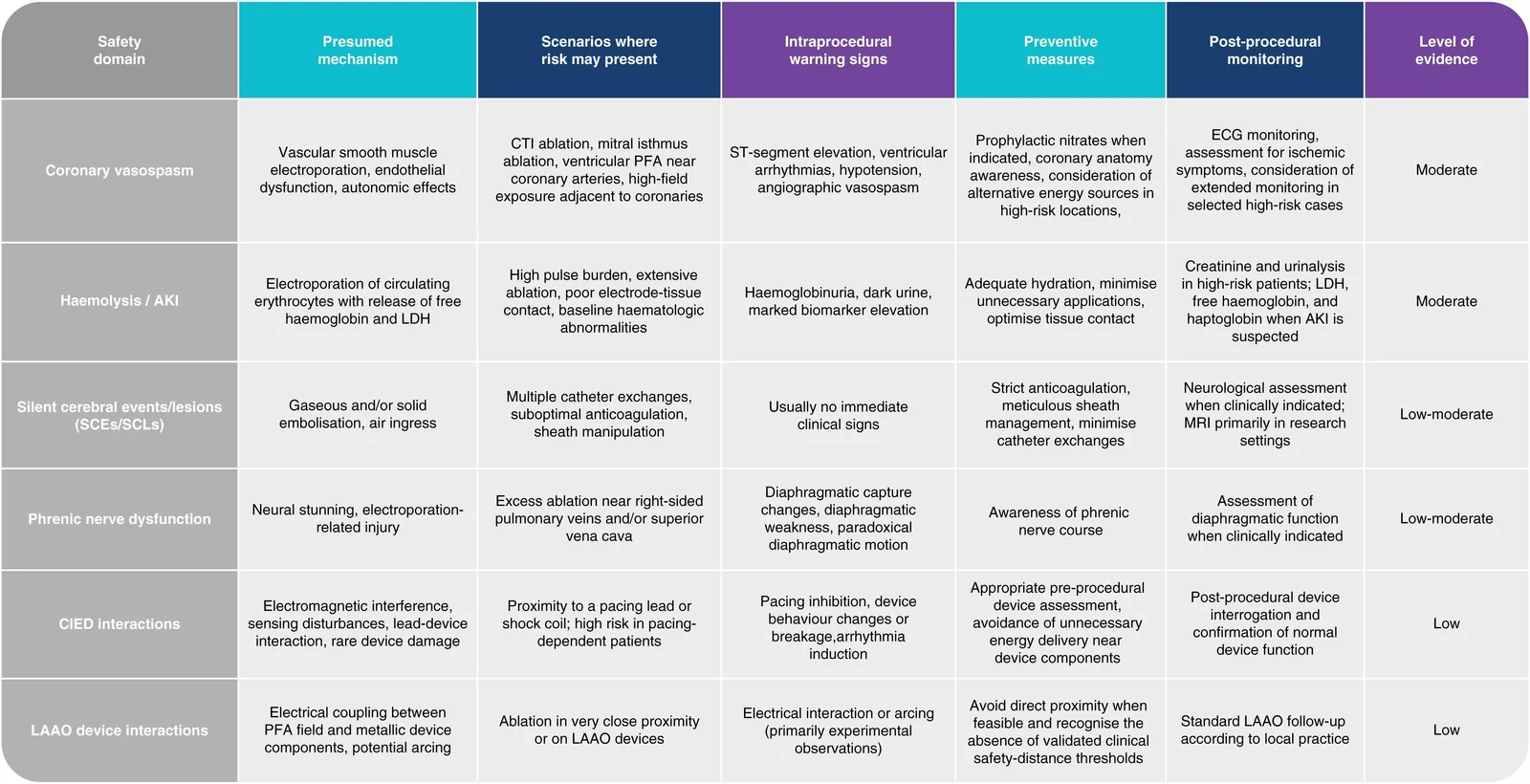
Safety profile of pulsed field ablation: mechanisms and clinical impact. Major safety domains associated with pulsed field ablation (PFA), their proposed mechanisms, and potential clinical consequences. The incidence and severity of these events may vary according to waveform characteristics, catheter design, polarity, lesion location, and procedural workflow. Accordingly, safety observations from individual PFA platforms should not be universally extrapolated to all current or future technologies.

## Conclusion

Current clinical experience with PFA highlights several reproducible physiologic patterns that shape its safety profile. Coronary vasospasm remains the most characteristic of these effects, ranging from subclinical vasoconstriction to rare episodes of ischaemia, arrhythmia, or delayed hemodynamic deterioration. Understanding coronary anatomy, optimizing lesion placement, and using preventive nitrates are the central strategies for reducing risk.

Haemolysis, a direct consequence of RBC electroporation, varies according to catheter design, PFA peak energy, and pulse burden. Although typically benign, progression to acute kidney injury can occur under high energy exposure or inadequate hydration, making dose limitation and fluid administration essential components of procedural planning.

Silent cerebral lesions reflect a combination of energy-related and procedural contributors, including gas formation, air ingress, catheter exchanges, and anticoagulation management. Their clinical implications remain uncertain, but technique-driven mitigation offers meaningful opportunities to reduce microembolic burden.

Across these domains, most physiologic effects are predictable and manageable, while serious complications remain uncommon. Continued refinement of technique and device design will further enhance procedural safety as PFA applications expand.

## Authors’ contributions

Final approval of the manuscript: all authors.

## Data Availability

The data underlying this article will be shared on reasonable request to the corresponding author.
